# Activated Cardiac Fibroblasts Control Contraction of Human Fibrotic Cardiac Microtissues by a β-Adrenoreceptor-Dependent Mechanism

**DOI:** 10.3390/cells9051270

**Published:** 2020-05-20

**Authors:** Przemysław Błyszczuk, Christian Zuppinger, Ana Costa, Daria Nurzynska, Franca Di Meglio, Mara Stellato, Irina Agarkova, Godfrey L. Smith, Oliver Distler, Gabriela Kania

**Affiliations:** 1 Center of Experimental Rheumatology, Department of Rheumatology, University Hospital Zurich, Wagistr. 14, 8952 Schlieren, Switzerland; Mara.Stellato@usz.ch (M.S.); Oliver.Distler@usz.ch (O.D.); 2 Department of Clinical Immunology, Jagiellonian University Medical College, 30-663 Cracow, Poland; 3 Department for BioMedical Research, Department of Cardiology, University Hospital Bern, 3008 Bern, Switzerland; christian.zuppinger@dbmr.unibe.ch; 4 Institute of Cardiovascular and Medical Sciences, University of Glasgow, Glasgow G12 8TA, UK; Ana.Costa@glasgow.ac.uk (A.C.); godfrey.smith@glasgow.ac.uk (G.S.); 5 Department of Public Health, University of Naples “Federico II”, 80131 Naples, Italy; dariaanna.nurzynska@unina.it (D.N.); franca.dimeglio@unina.it (F.D.M.); 6 InSphero, 8952 Schlieren, Switzerland; irina.agarkova@insphero.com

**Keywords:** cardiac microtissues, iPSC-derived cardiomyocytes, cardiac fibroblasts, cardiac fibrosis, cardiac rhythm, TGF-β signalling, drug screening, in vitro model

## Abstract

Cardiac fibrosis represents a serious clinical problem. Development of novel treatment strategies is currently restricted by the lack of the relevant experimental models in a human genetic context. In this study, we fabricated self-aggregating, scaffold-free, 3D cardiac microtissues using human inducible pluripotent stem cell (iPSC)-derived cardiomyocytes and human cardiac fibroblasts. Fibrotic condition was obtained by treatment of cardiac microtissues with profibrotic cytokine transforming growth factor β1 (TGF-β1), preactivation of foetal cardiac fibroblasts with TGF-β1, or by the use of cardiac fibroblasts obtained from heart failure patients. In our model, TGF-β1 effectively induced profibrotic changes in cardiac fibroblasts and in cardiac microtissues. Fibrotic phenotype of cardiac microtissues was inhibited by treatment with TGF-β-receptor type 1 inhibitor SD208 in a dose-dependent manner. We observed that fibrotic cardiac microtissues substantially increased the spontaneous beating rate by shortening the relaxation phase and showed a lower contraction amplitude. Instead, no changes in action potential profile were detected. Furthermore, we demonstrated that contraction of human cardiac microtissues could be modulated by direct electrical stimulation or treatment with the β-adrenergic receptor agonist isoproterenol. However, in the absence of exogenous agonists, the β-adrenoreceptor blocker nadolol decreased beating rate of fibrotic cardiac microtissues by prolonging relaxation time. Thus, our data suggest that in fibrosis, activated cardiac fibroblasts could promote cardiac contraction rate by a direct stimulation of β-adrenoreceptor signalling. In conclusion, a model of fibrotic cardiac microtissues can be used as a high-throughput model for drug testing and to study cellular and molecular mechanisms of cardiac fibrosis.

## 1. Introduction

Cardiac fibrosis refers to an excessive accumulation of stromal cells and extracellular matrix (ECM) proteins in the myocardium and represents a common pathophysiological scenario in a broad range of cardiovascular conditions, including myocardial infarction, hypertension, myocarditis, and hypertrophic or dilated cardiomyopathy [1]. Cardiac fibroblasts and myofibroblasts represent the most extensively characterised stromal cell types involved in fibrotic processes in the heart [2]. In the traditional view, resident cardiac fibroblasts become activated and overproduce ECM proteins such as collagen type I and III and fibronectin. Fibrogenesis is regulated by multiple profibrotic inputs, which activate a complex signalling network. In cardiac fibrosis, transforming growth factor β (TGF-β) is recognised as the key profibrotic cytokine activating quiescent cardiac fibroblasts. Constitutive overexpression of TGF-β was shown to induce interstitial cardiac fibrosis and cardiac hypertrophy in transgenic mice [3], whereas targeting TGF-β or its downstream mediators successfully reduced or prevented cardiac fibrosis in various animal models [4,5,6]. Progressive fibrosis causes tissue stiffening and may affect cardiac rhythm. Clinical data associate fibrotic heart condition with increased resting heart rate [7] and with elevated risk of developing life-threating arrhythmias [8,9]. The underlaying mechanisms remain obscure, however.

In the body, heart rhythm established by pacemaker cells located in the sinoatrial node of the heart is mainly under control of the autonomic nervous system. Sympathetic stimulation increases the rate, whereas parasympathetic stimulation decreases it. Neurotransmitter noradrenaline released by the sympathetic neurons increases heart rate by activating β-adrenergic receptors on cardiomyocytes. Under stress condition, β-adrenoreceptors are further activated by adrenaline—a catecholamine produced by the adrenal glands and released into the circulation. Binding of agonists to β-adrenergic receptors causes the formation of cyclic adenosine monophosphate and activation of protein kinase A that phosphorylates a number of proteins including myosin light chains and thus increases the ability of cardiomyocytes to relax following excitation contraction coupling [10]. In the heart, the increased relaxation is associated with increased rate and strength of contraction.

Because extracardiac stimuli are important regulators of the heart rhythm, it is difficult to study intracardiac mechanisms of cardiac contractility using in vivo systems only. Data from ex vivo and in vitro cardiac models may shed more light on specific aspects of intracardiac rhythm regulation. Disease models using human cells offer a natural advantage over animal models by providing a proper genetic and cellular context. In cardiovascular research, difficulties in obtaining and culturing contractile primary human cardiomyocytes limited the development of in vitro human models for many years. The human induced pluripotent stem cell (iPSC) technology opened new perspectives and became a commonly used, unlimited source of spontaneously beating cardiomyocytes for regenerative medicine, drug discovery and toxicity screening [11].

A lack of the physiologically relevant experimental setting is a major drawback of many in vitro models. In general, traditional two-dimensional (2D) cell culture systems poorly mirror the biomechanical and biochemical microenvironment of the tissue. Three-dimensional (3D) cell culture technologies, instead, much better reflect the organ complexity and represent an attractive alternative to the commonly used 2D models. 3D cell culture systems, called microtissues or organoids, include cellular models on various bioscaffolds, but also scaffold-free spheroids comprising of one or more cell types. In recent years, various types of cardiac microtissues have been successfully used predominantly in regenerative medicine, but also as platforms for drug screening or as disease models [12]. Self-aggregating, scaffold-free cardiac microtissues consisting of human iPSC-derived cardiomyocytes and human primary cardiac fibroblasts cultured in nonadherent plates represent a high-throughput in vitro model of human cardiac tissue. These spontaneously beating cardiac microtissues show typical structures including well-developed myofibrils and recapitulate cardiac functionality including responsiveness to electrical stimulation [13]. In this work, we aimed to address how fibrotic changes affect contractility of human cardiac microtissues. We found that profibrotic condition and, in particular, activation of cardiac fibroblasts increase beating rate of cardiac microtissues, and this effect is at least partially mediated by endogenous activation of β-adrenergic receptors. Furthermore, we demonstrated that human cardiac microtissues represent an easy-to-use, reproducible high-throughput method for small molecule screening.

## 2. Material and Methods

### 2.1. Cellular Sources

Frozen human iPSC-derived cardiomyocytes (>99% troponin-positive) were purchased from Cellular Dynamics International, and were used directly upon thawing. Foetal human cardiac fibroblasts were purchased from Sigma (Cell Applications) and cells from passages 10–15 (>99% vimentin-positive, >99% collagen I-positive and <5% positive for filamentous form of α-SMA) were used. Adult cardiac fibroblasts were isolated from cardiac tissue samples obtained from the left atria of hearts from the patients with end-stage heart failure due to ischemic heart disease undergoing heart transplantation and from the waste fragments of donor normal hearts that were trimmed off while adjusting atrium size and shape during transplantation. Specimens were collected without patient identifiers according to the protocol revised by the Ethics Committee of the University of Naples Federico II (approval number 79/18) and in conformity with the principles outlined in the Declaration of Helsinki. Patients characteristic is available in Supplementary Table S1.

### 2.2. Isolation of Human Adult Cardiac Fibroblasts

Adult cardiac fibroblasts were isolated as described previously [14]. Briefly, cardiac tissue samples were minced and then enzymatically disaggregated by incubation in 0.25% trypsin and 0.1% (*w*/*v*) collagenase II (both Sigma-Aldrich, Basel, Switzerland) for 30 min at 37 °C. The digestion was stopped by adding Hanks’ Balanced Salt solution supplemented with 10% foetal bovine serum (FBS, Gibco, Paislay, UK). The tissue was further disaggregated by pipetting; noncardiomyocyte cells were separated from debris and cardiomyocytes by sequential centrifugation and passage through 20 µm cell sieve. Fibroblasts were isolated from cell suspension by immunomagnetic cell sorting through positive selection with anti-fibroblast MicroBeads (MiltenyiBiotec, Bergisch Gladbach, Germany). Following selection, fibroblasts were cultured in Dulbecco’s Modified Eagle’s Medium (DMEM, Sigma, Basel, Switzerland) supplemented with 10% FBS, penicillin 10,000 U, and streptomycin 10 mg/mL (all from Sigma, Basel, Switzerland) at 37 °C in 5% CO_2_. The aCFs from patients with heart failure and unaffected donors were used individually. Cells from passages 4–6 were used.

### 2.3. Fabrication and Culture of Microtissues

Three types of microtissues containing: (1) iPSC-derived cardiomyocytes and fibroblasts mixed in ratio 4:1, (2) fibroblasts only, and (3) iPSC-derived cardiomyocytes only were generated by self-assembling in 96-well GravityTRAP plates (InSphero, Schlieren, Switzerland), and cultured as one microtissue per well. Accordingly, for generation of one microtissue, 5000 cells suspended in 70 µL maintenance medium were placed in the GravityTRAP plates for three days in the slanted position. Maintenance medium contained 2% FBS (Gibco, Paislay, UK), 50 µM phenylephrine hydrochloride (Sigma), 0.3 µM L-ascorbic acid (Sigma), 50 µM 2-mercaptoethanol (Gibco, Paislay, UK), 50 U/mL penicillin/streptomycin (Gibco, Paislay, UK) in high glucose DMEM (Sigma). Upon self-assembly, microtissues were further cultured in the GravityTRAP plates in the maintenance medium for 10 days. Microtissues were cultured under standard culture conditions (at 37 °C, 5% CO_2_ in a humidified incubator). Medium was changed at days 0, 2, 4, and 7. Fibrotic differentiation of cardiac microtissues was induced at day 0 with 10 ng/mL recombinant human TGF-β1 (Peprotech, London, UK) and TGF-βR1 was blocked with 10^−8^–10^−5^ g/mL SD208 (Tocris, Zug, Switzerland). To address β-adrenoceptor-dependent mechanisms, microtissues were treated with 10 nM isoproterenol (Tocris) and/or 1 µM nadolol (Sigma) 15–90 min prior to video recording. Controls received solvents only.

### 2.4. Live Imaging and Video Analysis

Randomly chosen live microtissues were analysed using microscopes equipped with a humidified chamber at 37 °C and 5% CO_2_. Pictures of microtissues were captured using the Live Cell Imaging System (Olympus, Shinjuku, Japan) and size of microtissues was measured with the Xcellence Pro software (Olympus) as area in pixels. Increase in size was calculated as the size of microtissue at day 10/size of microtissue at day 0. Videos of contracting microtissues were recorded using the AxioObserver Z1 (Zeiss, Hombrechtikon, Switzerland) and the ZEN software. Videos were processed using Fiji software and custom-made macro. Contraction analysis was performed using Fiji software and MUSCLEMOTION macro [15]. 

### 2.5. FluoVolt Measurements

Voltage experiments were conducted on day 11. The microtissues were washed in serum-free medium consisting of DMEM (Gibco, Paislay, UK) supplemented with 10 mM D-Galactose (Sigma) and 1 mM sodium pyruvate (Sigma). The microtissues were loaded with voltage-sensitive dye: 0.1% FluoVolt and 1% PowerLoad (ThermoFisher, Waltham, WA, USA) in the above listed serum-free medium for 25 min at 37 °C 5% CO_2_. The voltage-sensitive dye was removed by washing in serum-free medium, and the multiwell plate was placed in an environmentally controlled stage incubator (37 °C, 5% CO_2_, >75% humidity) in the CellOPTIQ^®^ platform (Clyde Biosciences, BioCity Scotland) 30 min before experimentation. The fluorescent signal was recorded with an excitation at 470 nm, and emitted light was collected from the entire microtissue using a 10× Fluor objective and the intensity recorded by a photomultiplier tube at 510–560 nm at 10 kHz. A 15 s recording was taken of each microtissue. Offline analysis was performed using CellOPTIQ^®^.

### 2.6. Electrical Pacing

Prior to experiment, microtissues were transferred into a glass bottom cell culture dish. Electrical field pacing of microtissues was performed using a modified glass bottom culture dish with inserted platinum wires attached to a MyoPacer (IonOptix, Westwood, MA, USA) set to a frequency of 3 Hz. Microtissues were recorded using the Eclipse TE2000-U microscope (Nikon, Tokyo, Japan) equipped with heating chamber, temperature controller (Ibidi, Graefelfing, Germany) and HeroBlack6 GoPro camera (Back-Bone Gear, Ontario, Canada).

### 2.7. Quantitative RT-PCR

For one sample, 12 microtissues were pooled. Total RNA was isolated using the Quick RNA Micro Kit (Zymo Research, Irvine, CA, USA). cDNAs were amplified using the cDNA Synthesis Kit (Roche). Gene expression was detected using oligonucleotides complementary to transcripts of the analysed genes and the GoTaq qPCR Master Mix (Promega, Dubendorf, Switzerland) using the 7500 Fast Real-Time PCR System (Applied Biosystems, Waltham, MA, USA). Oligonucleotide sequences are available in the Supplementary Table S2. Transcript levels of human RPLP0 were used as endogenous reference, and relative gene expression was analysed using the 2^−ΔΔCt^ method.

### 2.8. Immunocytochemistry

In total, 25–30 microtissues were pooled, fixed overnight with 4% paraformaldehyde and embedded in 2% agarose and in paraffin. Microtissue sections of 3–5 µm were cut (Sophistolab, Muttenz, Switzerland) and deparaffinised following standard methods. Sections were blocked with 10% goat serum (Vector Laboratories, Burlingame, CA, USA) and stained with primary/secondary antibodies: rabbit anti-human α-SMA (Sigma, dilution 1:750)/goat anti-mouse AP (Dako, Glostrup, Denmark, dilution 1:50), rabbit anti-human vimentin (Abcam, Cambridge, UK, dilution 1:250)/goat anti-rabbit (Vector Laboratories, Burlingame, CA, USA, dilution 1:200), rabbit anti-human periostin (Abcam, dilution 1:250)/Polymer anti-rabbit (Histofine, Nichirei Biosciences Inc. Tokyo, Japan), rabbit anti-human fibronectin (Abcam, dilution 1:100)/Polymer anti-rabbit (Histofine), rabbit anti-human Ki67 (Abcam, dilution 1:100)/goat anti-rabbit (Vector Laboratories, dilution 1:200), rabbit anti-human connexin 43 (Abcam, dilution 1:200)/goat anti-rabbit (Vector Laboratories, dilution 1:500), and rabbit anti-human troponin T (Origene Technologies, Rockville, Maryland, USA, dilution 1:2000)/Polymer anti-rabbit (Histofine). Detection was performed with the Bond Polymer HRP Refine Detection kit (Leica, Heerbrugg, Switzerland) according to the manufacturer’s guidelines for troponin T, and with DAB or Vector Red (Vector Laboratories) for all other antibodies. Nuclei were counterstained with haematoxylin (J.T.Backer, Gliwice, Poland). Direct Red Sirius Red (Sigma-Aldrich, Basel, Switzerland ) staining was used to detect collagen deposition. Stained sections were analysed using the Olympus DP80 microscope and the cellSens Standard imaging software (Olympus, Shinjuku, Japan). Intensity of immunopositive signals and nuclei counterstaining for each section were quantified using the ImageJ software. Staining intensity is presented in arbitrary units (AU) representing immunopositive signal intensity corrected by the counterstained nuclei value.

### 2.9. Procollagen Type I and IL-6 ELISA

Supernatants from individual microtissues were collected at day 10 and stored at −80 °C. Undiluted supernatants were analysed with the human Procollagen I alpha 1 DuoSet ELISA (R&D Systems, Abingdon, UK) and the human IL-6 DuoSet ELISA (R&D Systems) following manufacturer’s protocol. Optical density was measured with the Synergy microplate reader (Biotek, Winooski, Vermont, USA) and the Gen5 software.

### 2.10. Caspase 3/7 Activity

Supernatants containing one microtissue each were collected at day 10 and immediately measured for apoptosis with the Caspase-Glo 3/7 Assay (Promega, Dubendorf, Switzerland) following the manufacturer’s protocol. Luminescence was measured with the Synergy microplate reader (Biotek, Winooski, Vermont, USA) and the Gen5 software.

### 2.11. Western Blotting

Cellular proteins of foetal human cardiac fibroblasts were extracted with RIPA buffer (Sigma-Aldrich) supplemented with protease inhibitor cocktail (Complete ULTRA Tablets, Roche) and phosphatase inhibitors (PhosphoStop, Roche) from cultivated cells. Protein concentration was quantified by colorimetric bicinchoninic acid assay according to the manufacturer’s protocol (Thermo Fisher Scientific). SDS-PAGE electrophoresis and wet-transfer method were used to separate and transfer proteins on nitrocellulose membranes, followed by 45 min incubation in blocking solution (tris buffered saline, Tween-20 (TBST, Thermo Fisher Scientific) containing 5% skim milk powder (Becton Dickinson AG, Allschwil, Swizterland). Membranes were incubated overnight with the following primary antibodies: anti-αSMA (1:1000, A2547, clone 1A4, Sigma-Aldrich), anti-Fibronectin (1:2000, Abcam: ab2413) or GAPDH (1:10,000, clone 14C10, #2118, Cell Signalling). Horseradish peroxidase (HRP)-conjugated secondary antibodies (1:5000) were used for detection with ECL substrate (SuperSignal West Pico Plus, Thermo Fisher Scientific) and development on the Fusion Fx (Vilber, Collegien, France). Densitometric analyses were performed with ImageJ 1.47t. Fold changes were computed after normalization to GAPDH.

### 2.12. Cell Contraction Assay

To assess the contractile properties of foetal human cardiac fibroblasts, the Contraction Assay Kit (Cell Biolabs, San Diego, CA, USA) was used following the manufacturer’s protocol. Cells were cultivated with or without 10 ng/mL TGF-β for 72 h and reseeded in collagen gels for a further 72 h. Each condition was analysed in triplicates or quadruplicates. Images were taken at time 0, 24, 48 and 72 h after reseeding in a collagen gel. Areas of the gels were measured by ImageJ. Percentage of contraction of all conditions was measured compared to the average of unstimulated cells at day 0.

### 2.13. Statistics

Normally distributed data were analysed by two-tailed unpaired or paired Student’s *t*-test and unpaired two-tailed ANOVA followed by uncorrected Fisher’s LSD post-hoc test. All analyses were computed using GraphPad Prism 8 software. Differences were considered as statistically significant for *p* < 0.05.

## 3. Results

### 3.1. Cardiac Fibroblasts Improve Integrity and Contractility of Human Cardiac Microtissues

**Figure 1:**
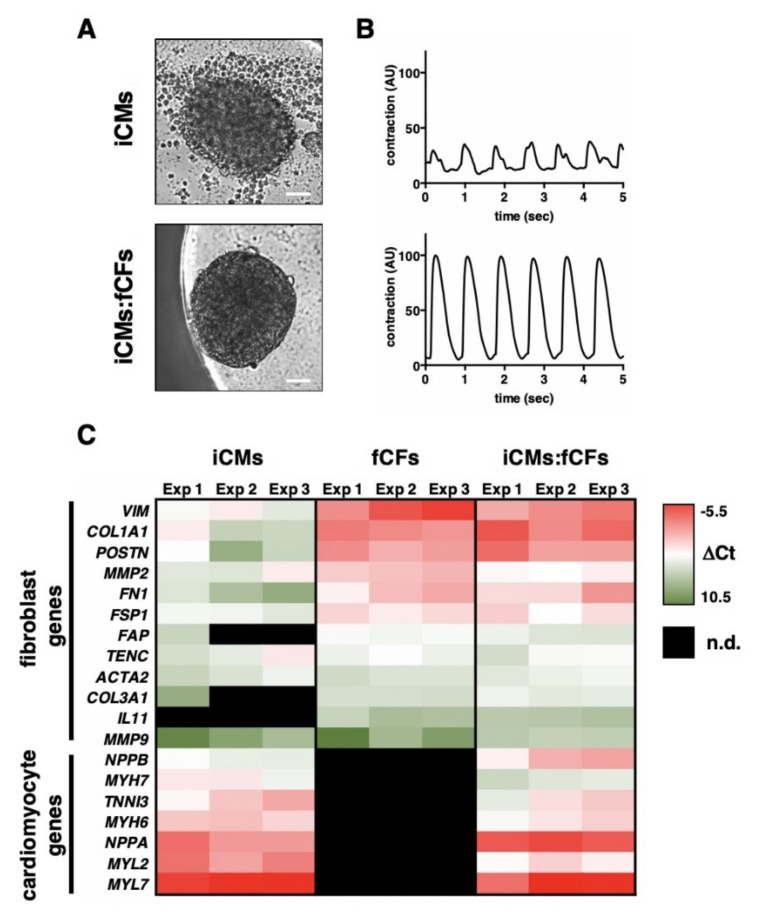
Characteristics of cardiac microtissues. Panel (**A**) illustrates typical morphologies (bar = 50 μm) and panel (**B**) shows typical contraction patterns of cardiac microtissues generated using iPSC-derived cardiomyocytes (iCMs) only (top) or iCMs mixed with foetal cardiac fibroblasts (fCFs) in a ratio of 4:1 (iCMs:fCFs, bottom). Representative contractions are available in the Supplementary Materials (Videos S1 and S2). A heat map in panel (**C**) indicates expression of cardiomyocyte and fibroblast genes in microtissues containing iCMs only (left), fCFs only (center) or iCMs:fCFs (right). Each segment indicates the average (*n* = 3–5) expression of one experiment. Lower −Δ C_t_ values indicate higher relative expression. n.d.—not detected.

Using human iPSC-derived cardiomyocytes (iCMs) and human foetal cardiac fibroblasts (fCFs), we developed a high throughput in vitro model of human cardiac tissue [16]. Cardiac microtissues consisting of 5000 cells each were generated by self-assembly (Figure S1). iCMs assembling without fibroblasts, however, formed loose cell aggregates (Figure 1A, Video S1). Addition of fCFs to iCMs in a ratio of 1:4 (fCFs:iCMs) allowed for formation of compact, spontaneously contracting cardiac microtissues (Figure 1A,B, Video S2) In the next step, we performed transcriptional profiling of microtissues by qPCR for genes characteristic for cardiomyocytes and fibroblasts. Seven genes characteristic for cardiomyocytes were detected in microtissues containing iCMs (iCM and iCM:fCF microtissues), but not in those generated with fCFs only. In contrast, nine out of twelve genes associated with fibroblasts and fibrosis were consistently detected in all types of microtissues (Figure 1C). However, expression levels of most of fibroblastic genes were substantially higher in fCFs than in iCMs microtissues. As expected, the expression profile of iCMs:fCFs microtissues showed high levels of all cardiac and fibroblastic genes (Figure 1C).

### 3.2. TGF-β1 Induces Fibrotic Changes in Human Cardiac Microtissues

**Figure 2:**
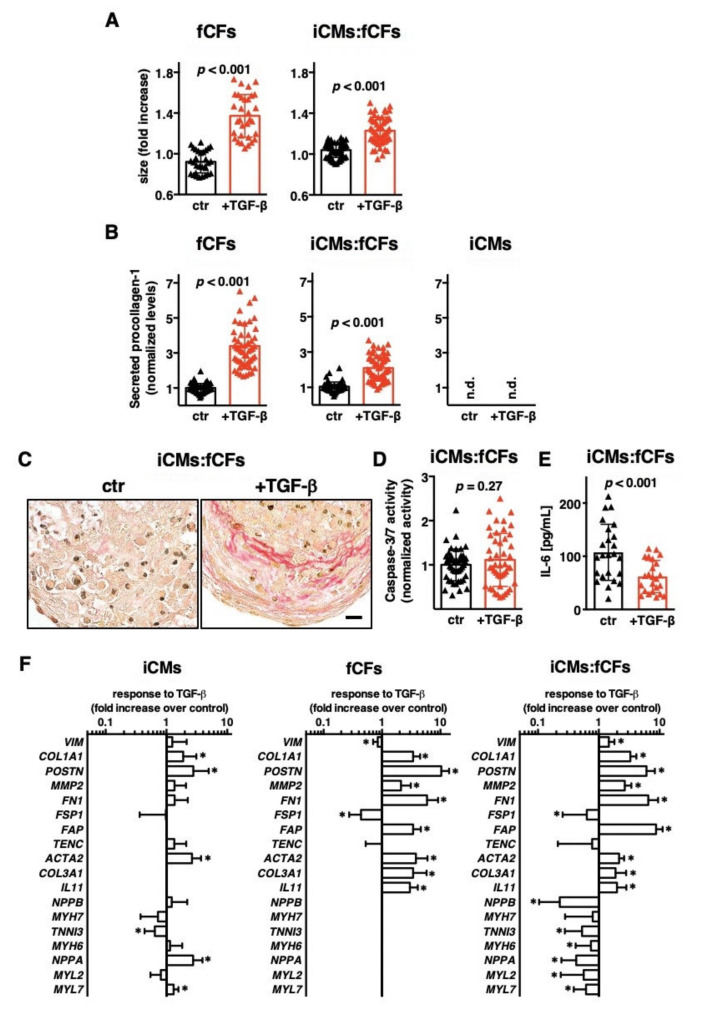
TGF-β1 activates foetal cardiac fibroblasts in microtissues. Panel (**A**) demonstrates changes in size of microtissues generated with fCFs only (fCFs, left) and iCMs mixed with fCFs in ratio 4:1 (iCMs:fCFs, right) cultured in the presence (red) or absence (black) of TGF-β1 (10 ng/mL) for 10 days. Panel (**B**) shows relative levels of procollagen I (measured by ELISA), at day 10 in supernatants of all three microtissue types: fCFs (left), iCMs:fCFs (middle) and iCMs (right). Graphs show cumulative data of 2–5 independent experiments. Each dot represents data of one microtissue. Panel (**C**) illustrates representative picrosirius red staining in iCMs:fCFs microtissues at day 10 (bar = 10 μm). Panel (**D**) shows caspase 3/7 activity measured at day 10 in iCMs:fCFs microtissues. Graphs show cumulative data of 3 independent experiments. Panel (**E**) shows IL-6 levels measured by ELISA, at day 10 in supernatants of iCMs:fCFs microtissues. Graphs show cumulative data of 3 independent experiments. Each triangle represents data of one microtissue. Panel (**F**) summarizes fold changes in gene expression in indicated microtissues in the presence of TGF-β1 (in relation to expression in the absence of TGF-β1). * *p* < 0.05. Graphs show cumulative data of 3–4 independent experiments, *n* = 11–20. For all graphs, *p* values were calculated with the Student’s *t*-test. n.d.—not detected.

TGF-β1 represents a potent inducer of profibrotic changes in cardiac fibroblasts. In the first step, we exposed microtissues to TGF-β1 for 10 days. In the presence of TGF-β1, both fCFs and iCMs:fCFs microtissues significantly increased their size (Figure 2A, Figure S2). 

In the next step, we measured procollagen type I secretion, which is an important hallmark of the ongoing fibrotic processes. As expected, fCFs microtissues produced markedly more collagen I upon TGF-β1 stimulation. Under this stimulatory condition, significant increase in procollagen type I production was also observed for iCMs:fCFs microtissues (Figure 2B). Importantly, iCMs microtissues did not produce detectable levels of procollagen I (Figure 2B) pointing to fibroblasts as the main source of collagen I in cardiac microtissues. Increased collagen production in iCMs:fCFs microtissues treated with TGF-β1 was confirmed by picrosirius red staining (Figure 2C). To address whether treatment with TGF-β1 induced apoptosis in this model, we measured caspase 3/7 activity in iCMs:fCFs microtissues. We observed similar caspase 3/7 activity in iCMs:fCFs microtissues treated with and without TGF-β1 (Figure 2D). Additionally, iCMs:fCFs microtissues treated with TGF-β1 produced significantly less IL-6 (Figure 2E).

Cardiac fibrogenesis is a transcriptionally regulated process. Upon TGF-β1 stimulation, genes characteristic for cardiomyocytes were only slightly dysregulated in iCMs microtissues. In iCMs:fCFs microtissues, instead, cardiomyocyte-specific genes were downregulated following TGF-β1 treatment (Figure 2F). Analysis of fibrotic genes showed that most of them were strongly upregulated in fCFs and iCMs:fCFs microtissues in response to TGF-β1 (Figure 2F).

**Figure 3:**
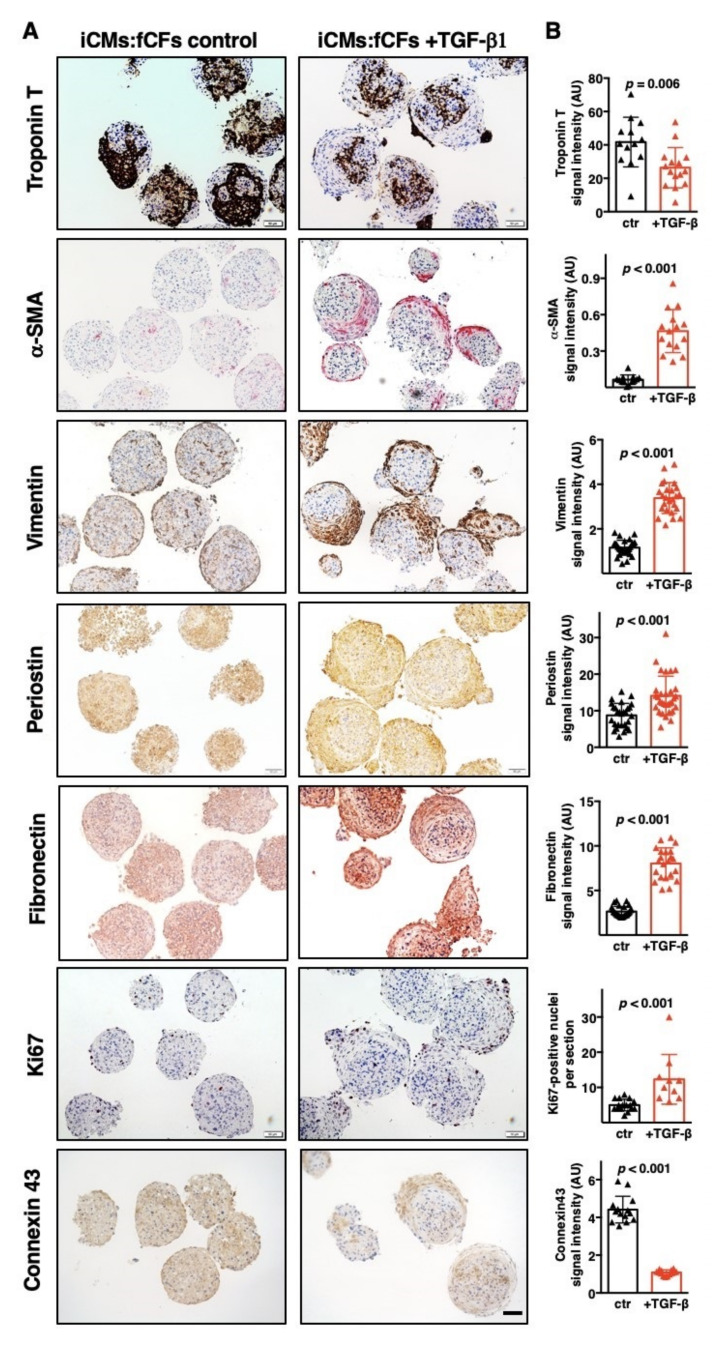
TGF-β1 induces fibrotic phenotype in cardiac microtissues. Immunohistochemistry of iCMs:fCFs microtissues cultured in the presence or absence of TGF-β1 (10 ng/mL) at day 10. Panel (**A**) illustrates representative staining for the indicated proteins at the indicated condition (bar = 50 μm). Higher magnification pictures are available in the Supplementary Materials (Figure S3). Panel (**B**) shows quantification of the respective staining for microtissues cultured in the presence (red) or absence (black) of TGF-β1. Graphs show cumulative data of 3 independent experiments. Each triangle represents data for one microtissue. *p* values were calculated with the Student’s *t*-test.

TGF-β1-induced changes in gene expression was followed by the analysis of the respective proteins in cardiac microtissues. To this aim, selected markers were analysed by immunohistochemistry in iCMs:fCFs microtissues cultured with or without TGF-β1. We found significantly less expression of the cardiac cell marker troponin T and gap junction protein connexin 43 in iCMs:fCFs microtissues exposed to TGF-β1. Instead, proteins produced by quiescent (vimentin, fibronectin) or activated (α-SMA, periostin) fibroblasts were more abundant in TGF-β1-treated cardiac microtissues. The number of proliferative (Ki67-positive) cells was also higher in iCMs:fCFs microtissues cultured with TGF-β1 (Figure 3, Figure S3). In conclusion, our results indicate that TGF-β1 effectively induced fibrogenesis in cardiac microtissues.

### 3.3. Pharmacological Targeting of TGF-βR1 Signalling Prevents from Fibrotic Changes in Human Cardiac Microtissues

**Figure 4:**
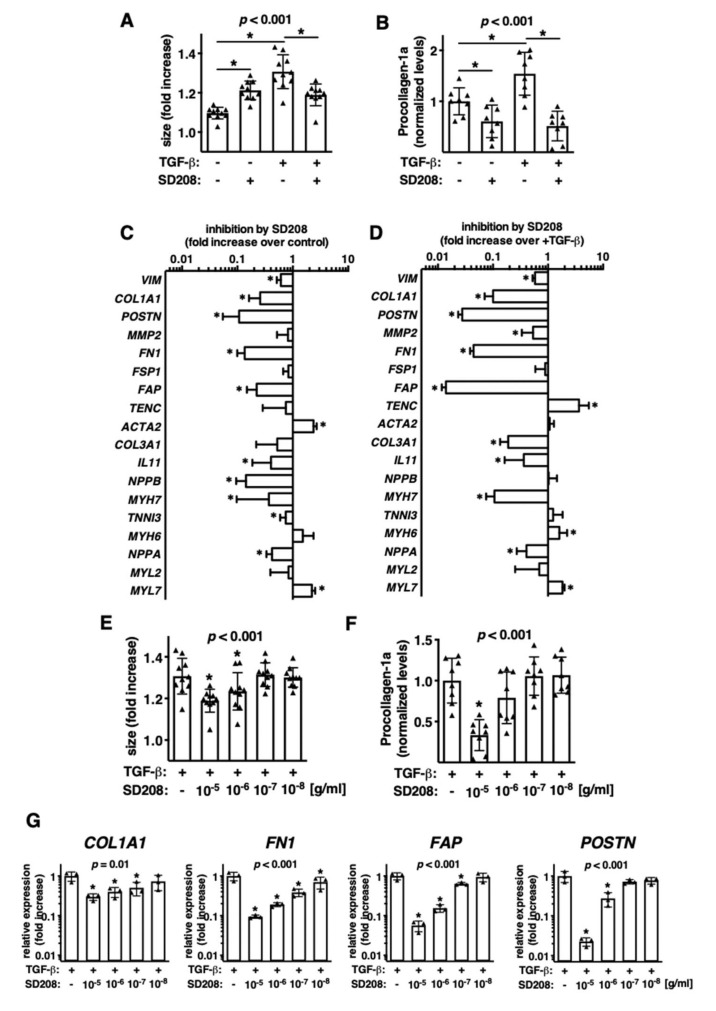
Pharmacological targeting of microtissues with TGF-βR1 inhibitor SD208. (**A**–**D**) iCMs:fCFs microtissues were cultured in the presence or absence of TGF-β1 (10 ng/mL) and SD208 (10 µg/mL). Panel (**A**) shows changes in size of microtissues and panel (**B**) normalized levels of secreted procollagen I (measured by ELISA in supernatants) at the indicated conditions at day 10. Each dot represents data for one microtissue. *p* values were calculated with ANOVA followed by uncorrected Fisher’s LSD tests for selected groups, * *p* < 0.05. Panels (**C**,**D**) show changes in gene expression in response to treatment with SD208 of microtissues cultured in the absence (**C**) or presence (**D**) of TGF-β1. *n* = 6, *p* values were calculated with the Student’s *t*-test, * *p* < 0.05. (**E**–**G**) iCMs:fCFs microtissues were cultured in the presence of TGF-β1 (10 ng/mL) and serial dilutions of SD208. Panel (**E**) shows changes in microtissue size, panel (**F**) normalized levels of secreted procollagen I and panel (**G**) relative expression of selected genes at the indicated conditions at day 10. Each dot represents data for one microtissue. Each triangle represents data for one microtissue. *p* values were calculated with ANOVA followed by uncorrected Fisher’s LSD tests versus +TGF-β group, * *p* < 0.05.

In the next step, we asked whether the fibrotic cardiac microtissue model is relevant to test anti-fibrotic compounds. To this aim, we used the TGF-βR1 inhibitor SD208 and tested it in the iCMs:fCFs microtissues cultured in the presence or absence of TGF-β. We observed that SD208 inhibitor effectively blocked TGF-β1-mediated microtissue growth (Figure 4A).

Furthermore, we found that SD208 suppressed procollagen type I production in iCMs:fCFs microtissues cultured not only with, but also without TGF-β1 (Figure 4B). Given the effect of the inhibitor in the absence of exogenous TGF-β1, we analysed gene expression in iCMs:fCFs microtissues cultured with and without SD208. Expression of 12 out of 18 analysed genes were significantly changed in response to SD208 treatment (Figure 4C). Similar results were obtained for treatment of iCMs:fCFs microtissues with SD208 in the presence of TGF-β1 (Figure 4D). Next, we analysed dose-dependent responses of microtissues to SD208. In these experiments, iCMs:fCFs microtissues were cultured in the presence of TGF-β1 and serial dilutions of SD208. Our results showed dose-dependent responses of iCMs:fCFs microtissues to SD208 in terms of microtissue size (Figure 4E), collagen type I secretion (Figure 4F) and expression of profibrotic genes (Figure 4G). Taken together, these data suggest that cardiac microtissues represent a useful model for pharmacological studies.

### 3.4. Activated Cardiac Fibroblasts Increase the Contraction Rate of Cardiac Microtissues

**Figure 5:**
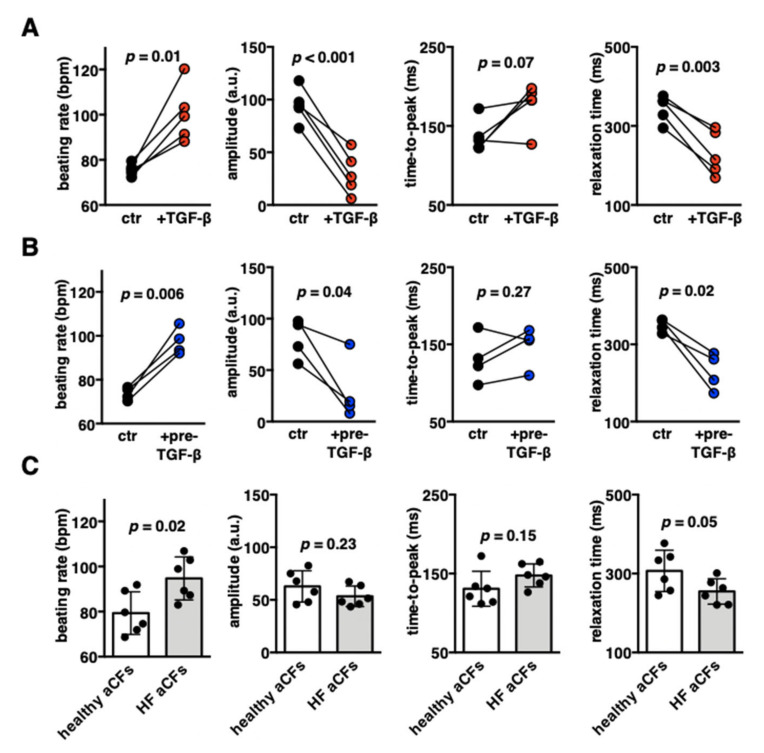
Contractile properties of cardiac microtissues containing foetal or adult cardiac fibroblasts. Panel (A) shows quantification of contraction parameters of iCMs:fCFs microtissues cultured in the presence (red) or absence (black) of TGF-β1 (10 ng/mL) at day 10. Quantification of contraction parameters of microtissues containing fCFs pretreated with TGF-β1 for 3 days prior microtissue formation (blue) or untreated fCFs (black) recorded at day 10 are shown in panel (B). Each dot represents average data of one experiment. Data of individual experiments are available in the Supplementary Materials (Figures S5 and S8). *p* values were calculated with the paired Student’s *t*-test. Panel (C) shows quantification of contraction parameters of cardiac microtissues containing aCFs from unaffected hearts (white) or heart failure (HF) patients (grey). Each dot represents average data of one experiment (*n* = 14–18). *p* values were calculated with the Student’s *t*-test. Representative contraction records are available in the Supplementary Materials (Figures S5, S10B and S11 and Videos S2–S6).

Contractility represents the main functional feature of cardiomyocytes. Using high-speed movies and motion-tracking analysis, we examined contractile properties of iCMs:fCFs microtissues at day 10. Fibrotic changes triggered by TGF-β1 substantially deteriorated the contraction pattern of iCMs:fCFs microtissues. We consistently observed significantly increased contraction rate and reduced contraction amplitudes of fibrotic microtissues in all experiments. A more detailed analysis of contraction pointed mainly to the shortening of relaxation phase in iCMs:fCFs microtissues upon treatment with TGF-β1 (Figure 5A, Figures S4 and S5, Video S3).

We hypothesized that TGF-β1 affected the contractility of iCMs:fCFs microtissues by activation of fCFs, rather than by a direct effect on iCMs. To validate this hypothesis, we generated cardiac microtissues using fCFs pretreated with TGF-β1 for three days (prior microtissue formation) and analysed their contractility in the absence of exogenous TGF-β1 at day 10. Pretreatment with TGF-β1 effectively activated fCFs as indicated by increased production of collagen, α-SMA and fibronectin in 2D cultures (Figure S6). We observed that iCMs:fCFs microtissues containing activated fCFs displayed increased size and secreted significantly more procollagen I (Figure S7), showed increased contraction rate, reduced contraction amplitudes and shortened relaxation phase (Figure 5B, Figures S4 and S8, Video S4), and successfully reproduced the contraction pattern observed in fibrotic (+TGF-β1) cardiac microtissues.

To further address the relevance of CFs to cardiac microtissue contractility, we compared cardiac microtissues containing adult CFs (aCFs) obtained from unaffected hearts of mostly young individuals (healthy aCFs) to aCFs from patients with heart failure (HF aCFs). Cardiac microtissues containing healthy aCFs and HF aCFs showed similar sizes at day 10 (Figures S9 and S10A). We found that iCMs:aCFs microtissues with HF aCFs showed increased contraction rate and shortened relaxation phase in comparison to cardiac microtissues containing healthy aCFs (Figure 5C, Figures S4 and S10B, Videos S5 and S6). Of note, microtissues containing healthy iCMs:aCFs more often responded to TGF-β1 stimulation by increasing contraction rate than those with HF aCFs (four of six vs. one of six, Figure S11). All these data indicate that contractile properties of cardiac microtissues depend on the activation status of CFs.

### 3.5. Unaffected Repolarization Phase in Fibrotic Cardiac Microtissues

**Figure 6:**
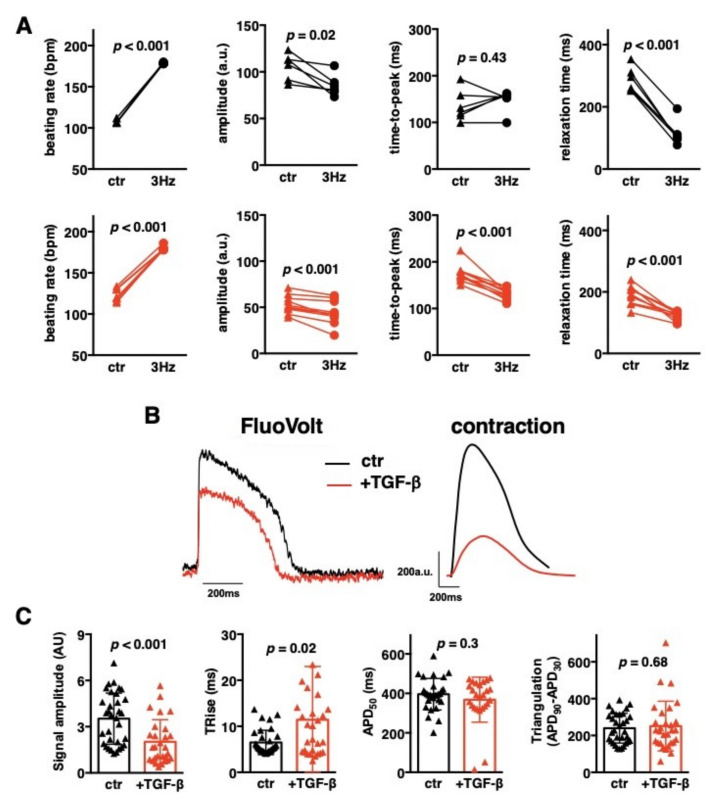
Cardiac microtissue electrophysiology. iCMs:fCFs microtissues were cultured in the presence (red) or absence (black) of TGF-β1 (10 ng/mL) for 10 days. Panel (**A**) shows quantifications of the contraction parameters recorded during spontaneous contractile activity (triangles, ctr) and then upon electrical stimulation with 3 Hz (circles, 3 Hz). Each dot represents data for one microtissue at the indicated condition, lines match data obtained from the same microtissue. *p* values were calculated with the paired Student’s *t*-test. Panel (**B**) illustrates representative action potentials recorded with FluoVolt probe (left) and the respective contractions (right). Quantifications of action potential parameters are shown in panel (**C**). Graphs show cumulative data of 3 independent experiments. Each dot represents data for one microtissue. *p* values were calculated with the Student’s *t*-test. APD—action potential duration, TRise—depolarisation phase.

In cardiomyocytes, mechanical contraction is triggered by electrical excitation. Next, we addressed the effect of external electrical stimulation of cardiac microtissues. Stimulation at 3 Hz induced a beating rate of 178–179 bpm in responsive microtissues, reduced contraction amplitude and substantially shortened contraction duration in both control and TGF-β1-treated iCMs:fCFs microtissues (Figure 6A, Videos S7 and S8), pointing to their full electrical responsiveness. Next, we analysed changes in membrane potential of control and fibrotic iCMs:fCFs microtissues using the relevant fluorescent probe (Figure 6B).

We found significantly lower action potential amplitudes in TGF-β1-treated iCMs:fCFs microtissues in comparison to controls (Figure 6C), suggesting reduced number of electrically active cells in fibrotic microtissues. Instead, there was no difference in the action potential duration, although treatment with TGF-β1 slightly prolonged the depolarization phase (Figure 6C). It seems that electrical activity of iCMs in cardiac microtissues was not significantly affected by profibrotic changes.

### 3.6. Endogenous β-Adrenergic Receptor Signalling Controls Increased Contraction Rate in Fibrotic Cardiac Microtissues

**Figure 7:**
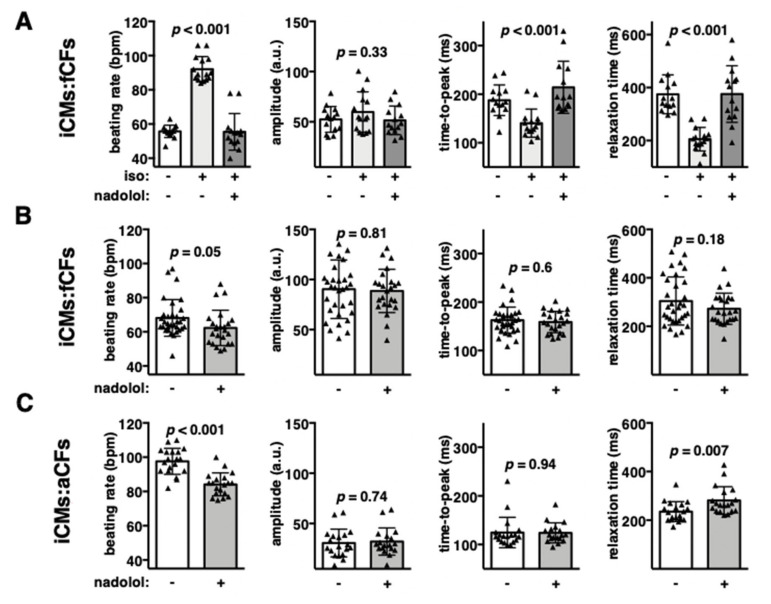
β-adrenergic receptor signalling in cardiac microtissues. Panel (**A**) shows quantification of contraction parameters of iCMs:fCFs microtissues stimulated with β-adrenoceptor agonist isoproterenol (iso, 10 nM) in the presence or absence of β-adrenoceptor blocker nadolol (1 µM). Unstimulated microtissues were used as controls. Graphs in panels (**B**,**C**) show contraction parameters of iCMs:fCFs (**B**) and iCMs:aCFs (**C**) microtissues treated with vehicle or 1 µM nadolol in the absence of isoproterenol. Each triangle represents data for one microtissue. *p* values were calculated with ANOVA for (**A**) and with the Student’s *t*-test for (**B**,**C**).

Action potential triggers cardiomyocyte contraction, but β-adrenoceptor-coupled mechanisms can modulate it. First, we used iCMs:fCFs and iCMs:aCFs microtissues and treated them with β-adrenoceptor agonist isoproterenol. As expected, stimulated microtissues showed increased contraction rate (Figure 7A, Figure S12). Isoproterenol significantly shortened both the contraction and relaxation phases of spontaneously contracting microtissues. In contrast, contraction amplitude remained unaffected. Addition of the β-adrenergic receptor blocker nadolol effectively prevented isoproterenol-induced changes in microtissue contraction (Figure 7A). These data confirmed functional β-adrenoceptors in our cardiac microtissues.

Next, we addressed the relevance of endogenous β-adrenoceptor signalling in cardiac microtissues. To this aim, we analysed the contractility of iCMs:fCFs and iCMs:aCFs microtissues in the presence and absence of nadolol. Treatment with nadolol only slightly reduced contraction of iCMs:fCFs microtissues (Figure 7B). In the next experiment, we used iCMs:aCFs microtissues showing high spontaneous contraction rate. In this model, nadolol significantly reduced contraction rate, whereas contraction amplitude remained unchanged (Figure 7C). We found that nadolol specifically affected relaxation, but not contraction phase. These results suggest activation of endogenous β-adrenoceptor signalling in iCMs:aCFs microtissues.

## 4. Discussion

In 3D microtissue models, fibroblasts have been recognized to improve microtissue architecture and biomechanics by providing proper ECM. We confirm that CFs significantly increased the integrity and synchronized contractility of human cardiac microtissues. As recently demonstrated, the use of a 4:1 ratio (iCMs:CFs) results in cardiac microtissues with stable phenotype and beating properties for up to one month [16]. Our data further demonstrated that contractility of these cardiac microtissues was substantially affected under profibrotic condition (i.e., in the presence of TGF-β1). TGF-β1 is a profibrotic cytokine that enhances fibroblast proliferation and production of ECM proteins. In our model, TGF-β1 induced fibrotic phenotype in cardiac microtissues, as indicated by elevated levels of fibrotic genes, secretion of procollagen I and fCFs proliferation. Previously, exogenous TGF-β1 has been shown to induce fibrosis in rat [17,18] or human [19] cardiac microtissue models. Furthermore, fibrotic phenotypes in cardiac microtissues or in biowire were also produced by collagen/fibroblast enrichment [20,21] or by constitutive activation of profibrotic pathways [22].

Our data demonstrated that pretreatment of fCFs with TGF-β1 prior to microtissue formation resulted in a similar effect on microtissue contractility as continuous stimulation with TGF-β1. This result suggested that activation of CFs, rather than a direct effect of TGF-β1 on iCMs, was a main mechanism responsible for deteriorated contraction of fibrotic cardiac microtissues. This idea was further strengthened by the observation that aCFs induced a contraction pattern (high contraction rate with low amplitude) of cardiac microtissues similar to those containing activated fCFs. CFs become activated following heart failure and with aging [23]. We observed that cardiac microtissues containing aCFs obtained from aged heart failure patients showed significantly higher beating rate than microtissues containing aCFs from young, healthy hearts. All these data suggested that activation status of CFs was a key factor determining contraction pattern of cardiac microtissues.

Fibrotic condition in the heart is associated with arrhythmias. Published data showed that activated rodent CFs could cause asynchronous contraction [18] and induce proarrhythmic changes in cardiomyocytes by affecting action potential profile [22,24]. In our model, fibrotic microtissues showed arrhythmic contractions only occasionally and their action potential profile remained unaffected. Instead, the fibrotic condition was associated with reduced action potential signal amplitude, suggesting a lower number of electrically active iCMs in these microtissues. Impaired conduction and contraction of these microtissues might be a consequence of iCMs decoupling and coupling of iCMs with CFs, reduced levels of gap junction protein connexin 43 and/or overproduction of cytokines and ECM by activated CFs. Secreted factors by activated CFs, biomechanical signalling through excessive ECM deposition and remodelling of mechanical junctions are known factors disturbing proper propagation of electrical impulses [25]. Signal conduction abnormalities, such as re-entry, can cause cardiac arrhythmias and may have serious clinical implications for patients with fibrotic hearts [9]. Clinical data suggest that ventricular fibrosis represents a strong predictor of life-threatening ventricular arrhythmia and sudden cardiac death in ischemic and nonischemic heart disorders [8].

In our model, profibrotic condition was consistently associated with increased beating rate of cardiac microtissues. Similar data were obtained in human cardiac microtissues containing mesenchymal stem cells instead of CFs following treatment with TGF-β1 [19]. Physiologically, increased beating rate of the heart is achieved by activation of β-adrenoreceptors. In normal adult cardiomyocytes, β1-adrenoreceptors are dominant, whereas iCMs contractility can be regulated by both β1- and β2-adrenoreceptors [26]. Previous data showed that the beating rate of human iCMs could be increased by treatment with the β-adrenergic agonist isoproterenol [26,27]. In line with these data, we could demonstrate the responsiveness of human cardiac microtissues containing iCMs to isoproterenol. In case of cardiac microtissues showing high beating rate (due to presence of activated aCFs), blockade of β-adrenergic receptors in the absence of exogenous agonists significantly decreased frequency. This finding indicated that activated CFs triggered endogenous β-adrenoreceptor signalling in cardiac microtissues. Mechanistically, stimulation of β-adrenoreceptors increases beating rate by enhancing cardiomyocyte relaxation [10]. Indeed, we observed that the reduced beating rate observed in the presence of β-adrenoreceptor blockers was associated with longer relaxation time. Accordingly, increased contraction rate of fibrotic cardiac microtissues correlated with shortened relaxation time. These results may suggest that activated CFs produce β-adrenergic agonists. Alternatively, fibrotic condition may sensitize β-adrenoreceptors. Cellular response to β-adrenergic stimulations is further modulated by multiple factors, such as cell surface receptor density, activity of the downstream signalling pathway, or by specific costimulations [28]. For example, fibrotic hearts of TGF-β-overexpressing mice showed increased density of β-adrenoreceptors on cardiomyocytes and altered β-adrenergic signalling [3]. Furthermore, collagen receptor β_1_-integrin [29], connective tissue growth factor [30] or tissue stiffness [31] have also been shown to regulate β-adrenergic signalling.

A successful preclinical platform has to combine physiological-like relevance with the high-throughput processing. Our fibrotic cardiac microtissue model is established in a 96-well format and certain parameters, such as size, contractile properties, changes in membrane potential, procollagen-I production or apoptosis can be measured for individual microtissues. In particular, recently developed software measuring the contractile activity of the whole microtissue allows for quick and easy analysis of the key properties of beating cardiac microtissues [15]. In our experiments, transcriptomic data were obtained from 10–12 pooled microtissues, but we used a simple RT-PCR to analyse gene expression. Application of the next-generation sequencing technology would allow obtaining transcriptomic data from one microtissue. Thus, this model could be used as a high-throughput platform for pharmacological screening with a timeframe for up to at least 10 days. The proof-of-concept experiments with the TGF-βR1 inhibitor SD208 confirmed that fibrotic cardiac microtissues represent a robust model for drug screening.

Although our human cardiac microtissue-based fibrotic platform combines technical ease, high throughput and reproducibility with physiological-like relevance, it also needs to be discussed in the context of its limitations. iCMs are characterized by their foetal developmental status [32]. Currently, there is no satisfactory alternative to iCMs. The usefulness of adult primary human cardiomyocytes is limited, as these cells contract upon chemical or electrical stimulation only [33]. Despite recent advances in the maturation of iCMs through improved biomechanics and cocultures with other cell types, so far, their maturation status does not reflect adult cardiomyocytes [13,34,35,36]. Another limitation of our model is the heterogeneity of iCMs. Conventional differentiation of iPSCs into iCMs results in populations of pacemaker, ventricular-like, atrial-like cells as well as nonbeating progenitors. The use of defined types of differentiated cardiomyocytes could further improve the relevance of the cardiac microtissue model. Moreover, addition of other cell types, such as endothelial cells or macrophages, to iCMs and fCFs might result in formation of microtissues that better mimic the structure and function of human cardiac tissue.

## Supplementary Materials

The following are available online at https://www.mdpi.com/2073-4409/9/5/1270/s1, Figure S1: Schematic presentation of experimental setup with time lines, Figure S2: Kinetics of microtissue growths, Figure S3: TGF-β1 induces fibrotic phenotypes in cardiac microtissues, Figure S4: Contraction patterns of cardiac microtissues containing foetal or adult cardiac fibroblasts, Figure S5: Effect of exogenous TGF-β1 on contractility of cardiac microtissues - individual experiments, Figure S6: Activation of foetal cardiac fibroblasts with TGF-β1, Figure S7: Effect of activated foetal cardiac fibroblasts on size and procollagen I secretion, Figure S8: Effect of activated foetal cardiac fibroblasts on contractility of cardiac microtissues - individual experiments, Figure S9: Size of cardiac microtissues containing adult cardiac fibroblasts, Figure S10: Size and contractile properties of cardiac microtissues containing adult cardiac fibroblasts, Figure S11: Effect of exogenous TGF-β1 on contractility of healthy iCMs:aCFs and HF iCMs:aCFs microtissues, Figure S12: b-adrenergic receptor stimulation of iCMs:aCFs microtissues; Table S1: Clinical characteristics of patients from which aCFs were obtained, Table S2: Primers used for quantitative RT-PCR; Video S1: Control iCMs only, Video S2: iCMs_fCFs control d10, Video S3: iCMSfCFs TGF-β1, Video S4: iCMsfCFs pre-TGF-β-3d_d10 D10, Video S5: iCMs_healthy aCFs control d10, Video S6: iCMS_HF aCMs control d10, Video S7: iCMs_fCFs control, Video S8: iCMs_fCFs 3Hz.
