# Supplementary material for: Activated Cardiac Fibroblasts Control Contraction of Human Fibrotic Cardiac Microtissues by a β-Adrenoreceptor-Dependent Mechanism
Source: Cells. 2020 May 20;9(5):1270. doi: 10.3390/cells9051270 (PMC7290967; doi:10.3390/cells9051270)
Supplement: Supplementary file 1 [file cells-09-01270-s001.zip › cells-787536-suppl-final/Blyszczuk et al, Cells-787536_Supplementary material and Figures_final.pdf]

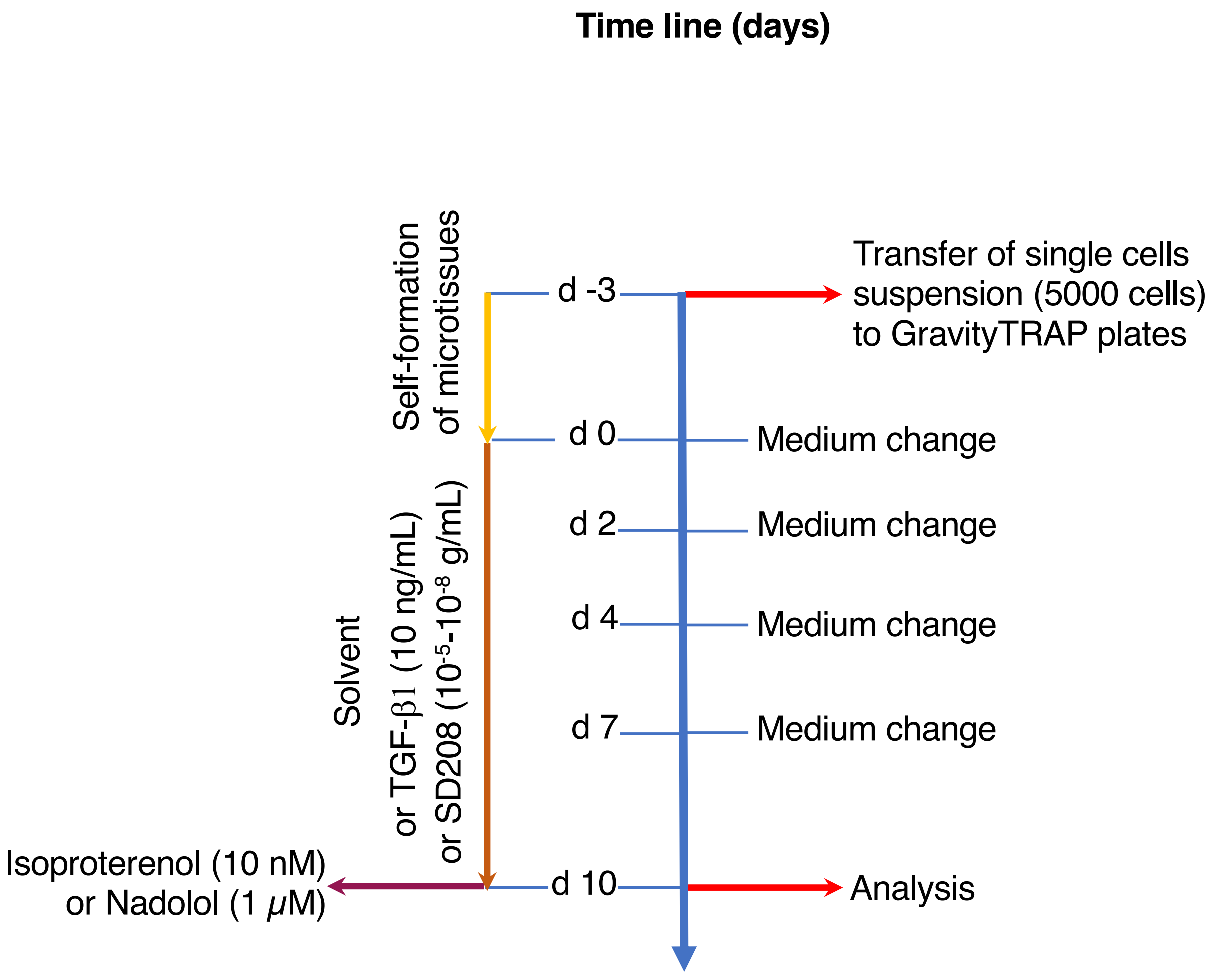

Supplementary figure 1. Schematic presentation of experimental setup with time lines. Transfer of single cell suspension (5000 cells) to in the GravityTRAP plates was done to form microtissue 3 days before the stimulation (d -3). Upon self-assembly, microtissues were further cultured in the GravityTRAP plates in the maintenance medium for 10 days (from d 0 until d 10). Medium was changed at days 0, 2, 4 and 7. Differentiation was induced at day 0 with 10 ng/mL recombinant human TGF-β1 and TGF-βR1 was blocked with 10^{-5}-10^{-8} g/mL SD208. To address β-adrenoceptor-dependent mechanisms, microtissues were treated with 10 nM isoproterenol and/or 1 µM nadolol, 15-90 minutes prior to video recording. Controls received solvents only.

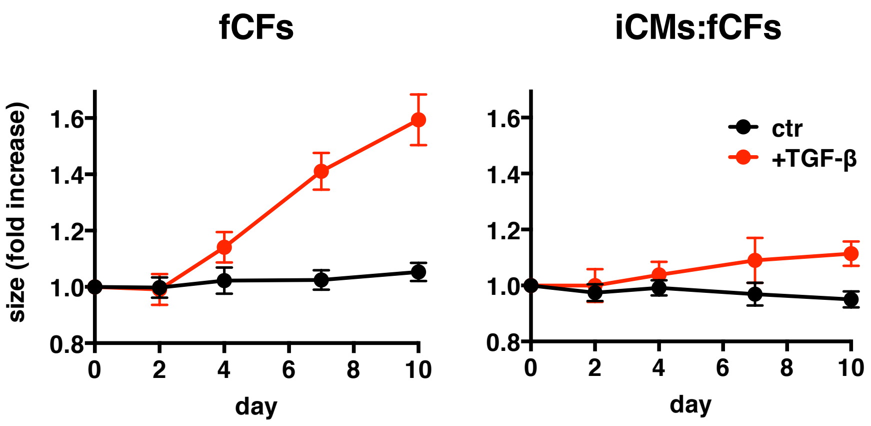

Supplementary Figure 2. Kinetics of microtissue growths. Changes in size of the fCFs (left) and iCMs:fCFs (right) microtissues in the presence (red) or absence (black) of TGF-β1. Increase in size of an individual microtissue was calculated as the size of microtissue at day 10/size of microtissue at day 0. n=10-11, each point indicates average ±SD.

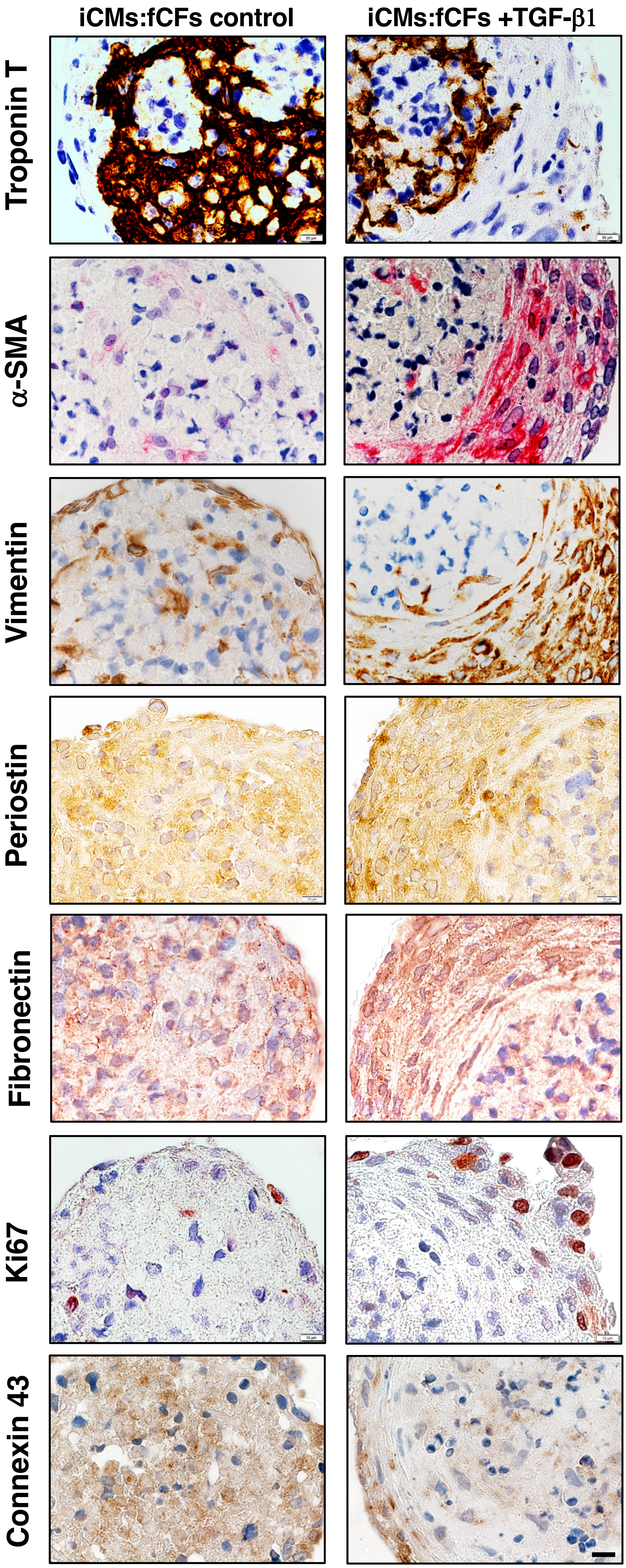

Supp. Figure 3

Supplementary Figure 3. TGF-β1 induces fibrotic phenotypes in cardiac microtissues. Immunohistochemistry of iCMs:fCFs microtissues cultured in the presence or absence of TGF-β1 (10 ng/mL) at day 10. The figure shows representative staining for the indicated proteins at high magnification (bar = 10 μm). Low magnification pictures and quantifications are presented in the Figure 3.

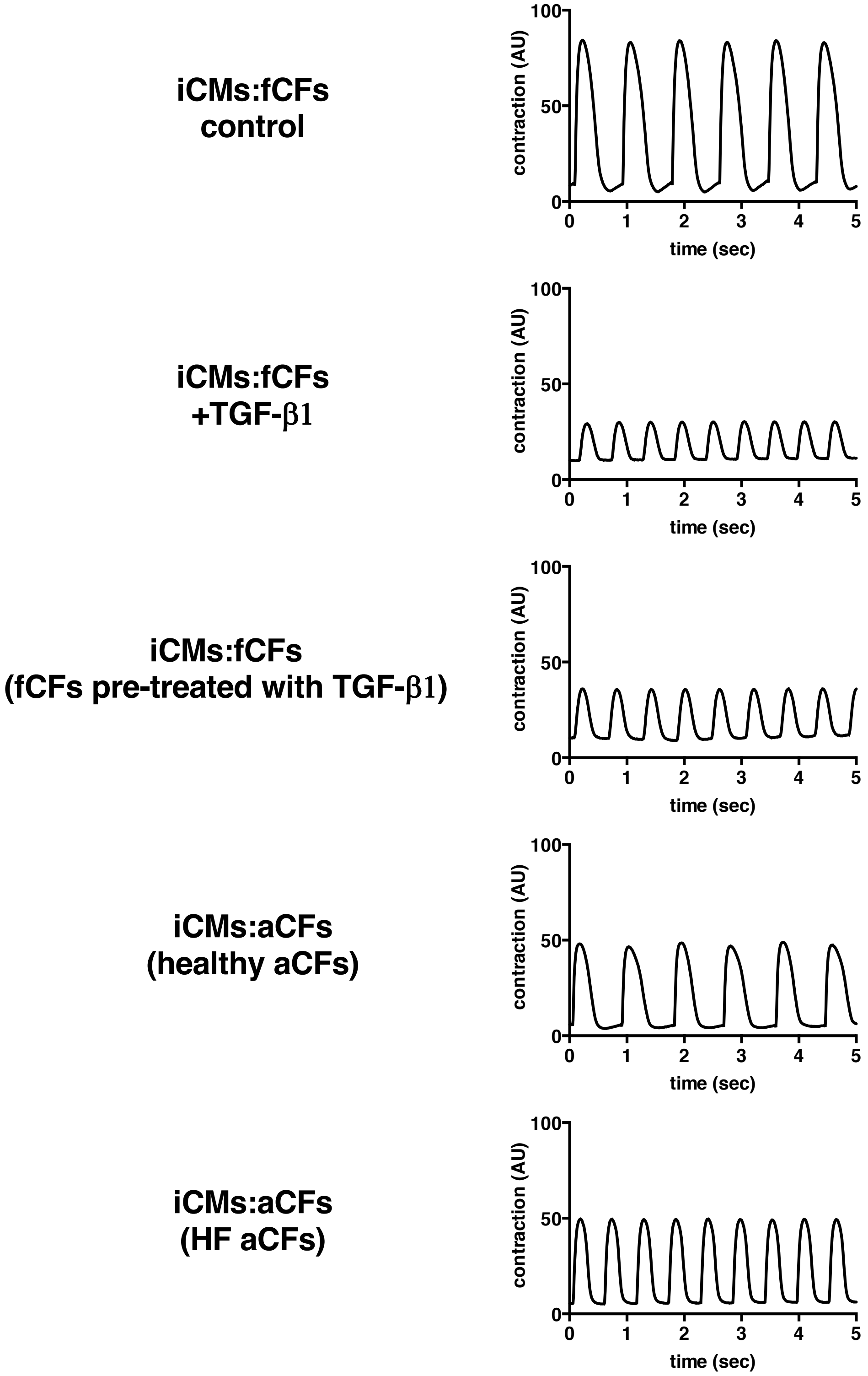

Supplementary Figure 4. Contraction patterns of cardiac microtissues containing foetal or adult cardiac fibroblasts. Graphs illustrate representative contraction patterns of cardiac microtissues generated using indicated CFs and the indicated conditions. Quantifications of the respective contractions are available in the Figure 5 and Suppl. Fig. 6 and 8.

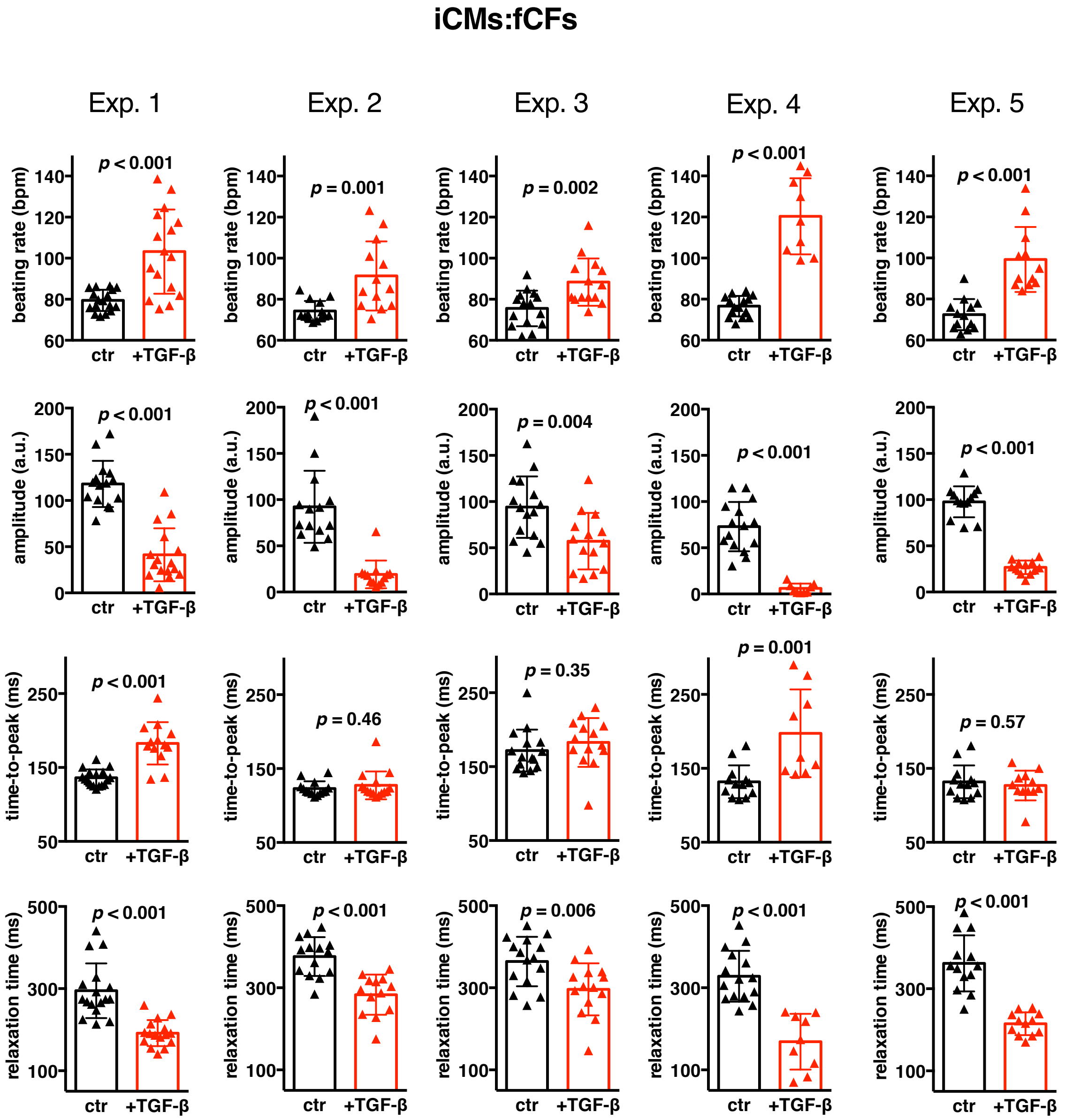

Supplementary Figure 5. Effect of exogenous TGF-β1 on contractility of cardiac microtissues - individual experiments. Figure shows quantification of contraction parameters of iCMs:fCFs microtissues cultured in the presence (red) or absence (black) of TGF-β1 (10 ng/mL) at day 10 obtained in independent experiments. Each dot represents data for one microtissue. *p* values were calculated with the Student's *t*-test.

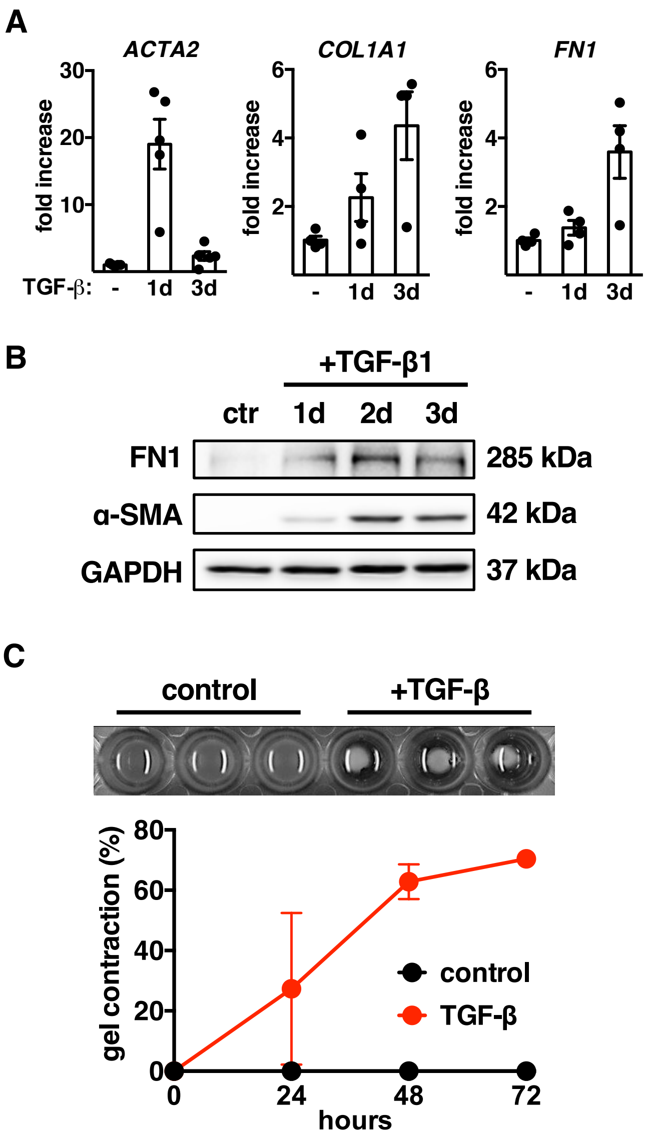

Supplementary Figure 6. Activation of foetal cardiac fibroblasts with TGF-β1. Panel **(A)** shows expression of indicated genes and panel **(B)** illustrates representative immunoblots of profibrotic proteins in fCFs in response to treatment with TGF-β1 (10 ng/mL). Panel **(C)** shows contraction of TGF-β1-pre-treated (red) and control (black) fCFs in 3D collagen matrix after indicated time. fCFs were pre-treated with TGF-β1 (10 ng/mL) for 3 days prior use. *ACTA2* – alpha smooth muscle actin; *COL1A1* – collagen I; *FN1* – fibronectin.

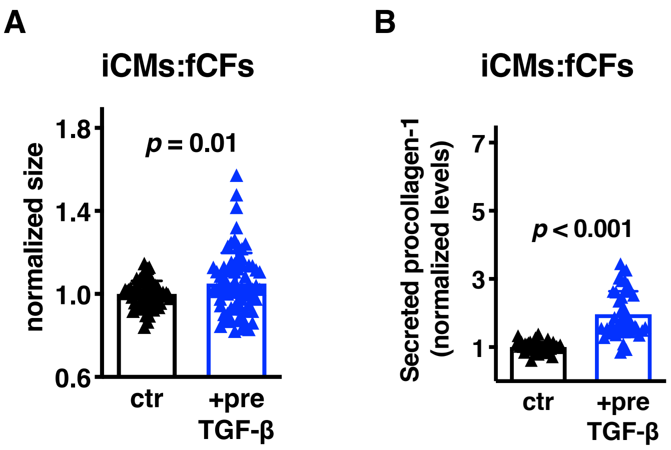

Supplementary Figure 7. Effect of activated foetal cardiac fibroblasts on size and pro-collagen I secretion. Panel **(A)** demonstrates changes in size of iCMs:fCFs microtissues containing fCFs pre-treated with TGF-β1 (10 ng/mL) for 3 days prior microtissue formation (blue) or untreated fCFs (black) recorded at day 10. Panel **(B)** shows relative levels of pro-collagen I, measured by ELISA, at day 10 in supernatants of iCMs:fCFs microtissues containing fCFs pre-treated with TGF-β1 (10 ng/mL) for 3 days prior microtissue formation (blue) or untreated fCFs (black) recorded at day 10. Graphs show cumulative data of 3 independent experiments. Each dot represents data of one microtissue Each triangle represents data for one microtissue. *p* values were calculated with the Student's *t*-test.

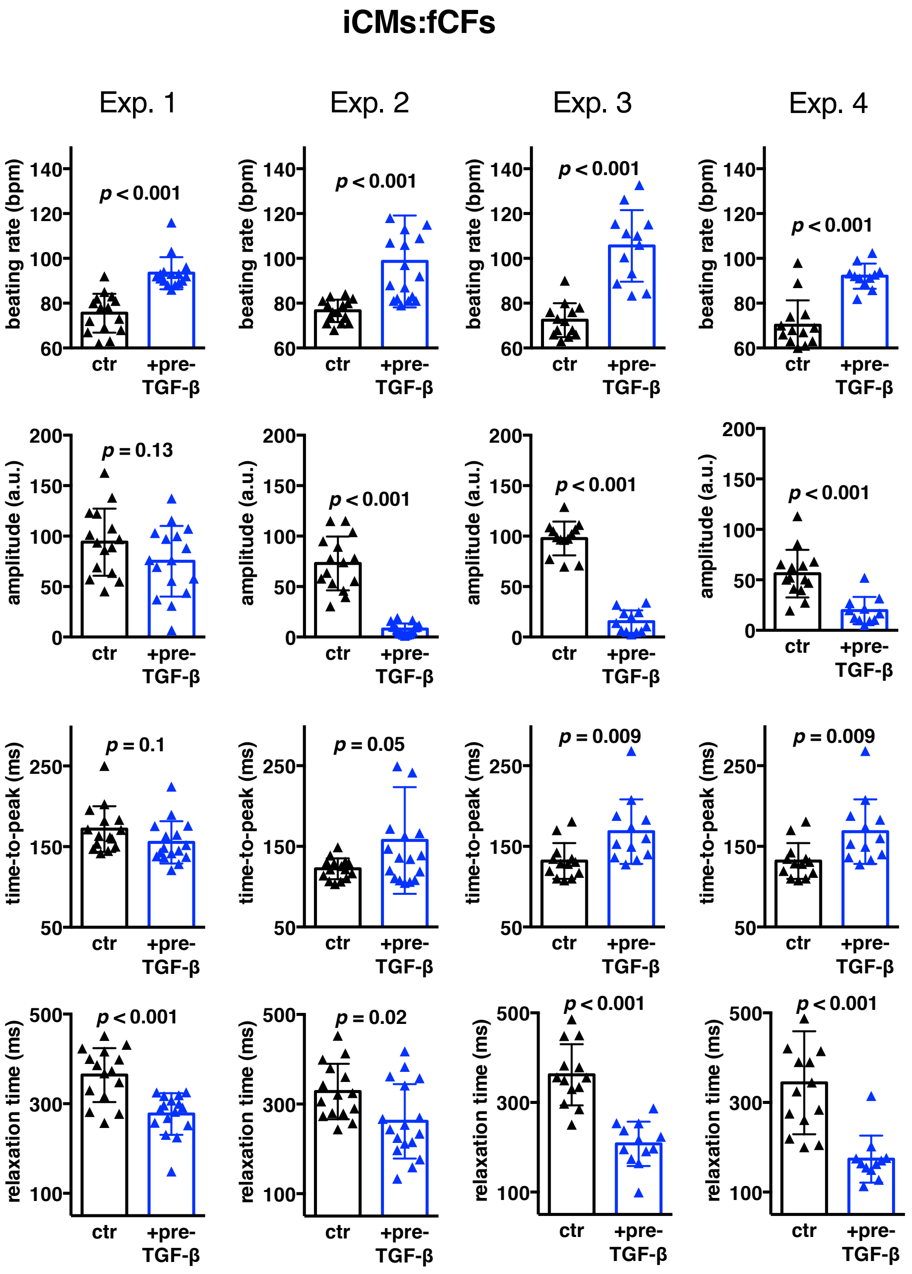

Supplementary Figure 8. Effect of activated foetal cardiac fibroblasts on contractility of cardiac microtissues - individual experiments. Figure shows quantification of contraction parameters of iCMs:fCFs microtissues containing fCFs pre-treated with TGF-β1 (10 ng/mL) for 3 days prior microtissue formation (blue) or untreated fCFs (black) recorded at day 10 obtained in independent experiments. Each dot represents data for one microtissue. p values were calculated with the Student's t-test.

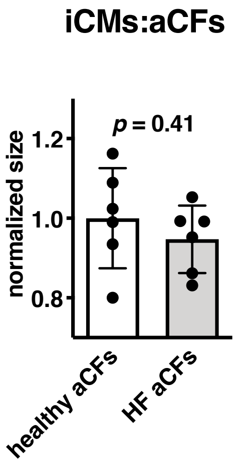

Supplementary Figure 9. Size of cardiac microtissues containing adult cardiac fibroblasts. Panel demonstrates changes in size of healthy iCMs:aCFs (white) and HF iCMs:aCFs (light grey) microtissues at day 10. Each dot represents data for one microtissue. *p* values were calculated with the Student's *t*-test.

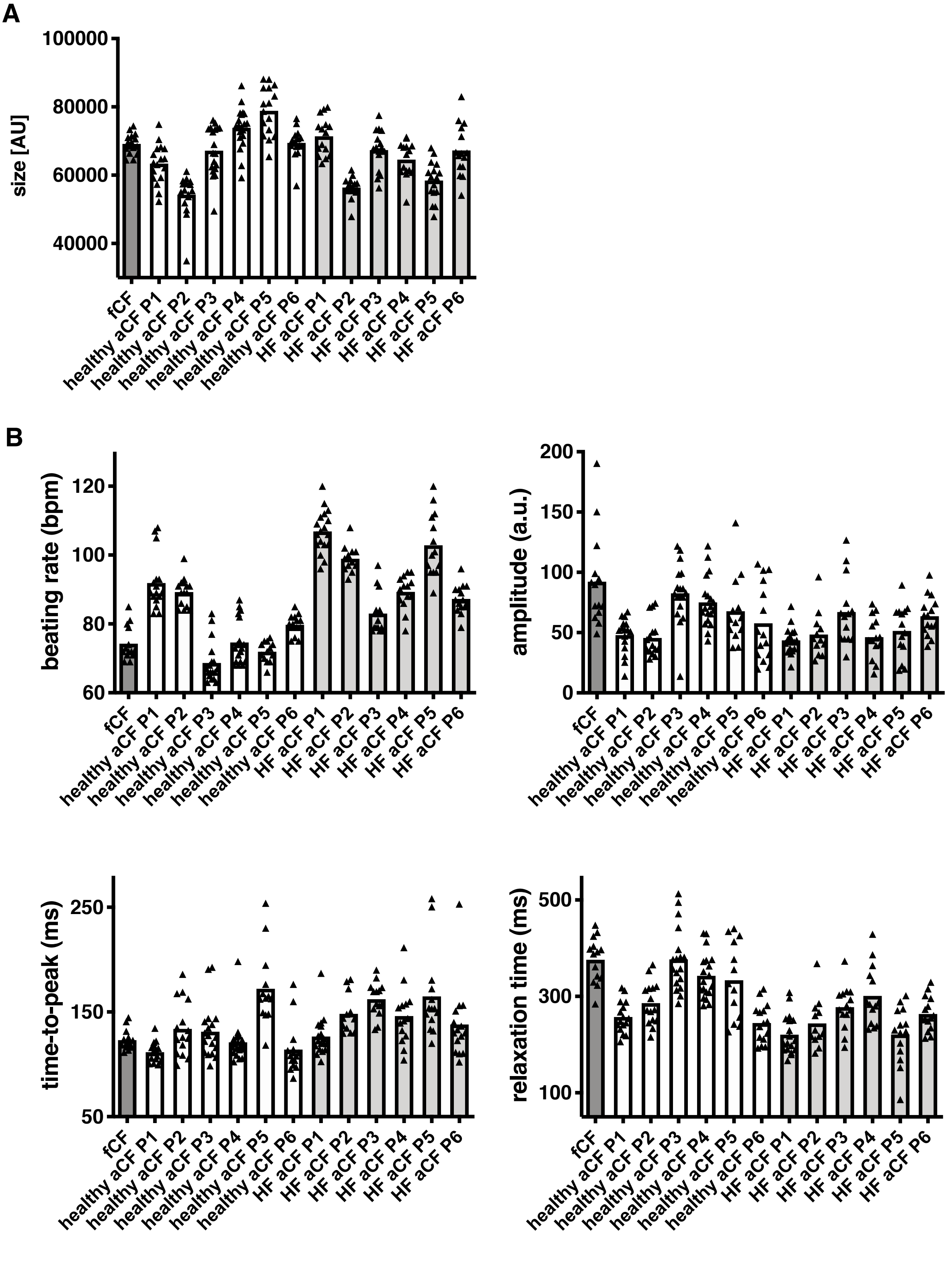

Supplementary Figure 10. Size and contractile properties of cardiac microtissues containing adult cardiac fibroblasts. Panel **(A)** demonstrates changes in size (showed as arbitrary unit: AU) of iCMs:fCFs (dark grey), healthy iCMs:aCFs (white) and HF iCMs:aCFs (light grey) microtissues at day 10. P1-P6 indicate aCFs from individual patients. Panel **(B)** shows quantification of contraction parameters of of iCMs:fCFs (dark grey), healthy iCMs:aCFs (white) and HF iCMs:aCFs (light grey) microtissues at day 10. P1-P6 indicate aCFs from individual patients. Each dot represents data for one microtissue.

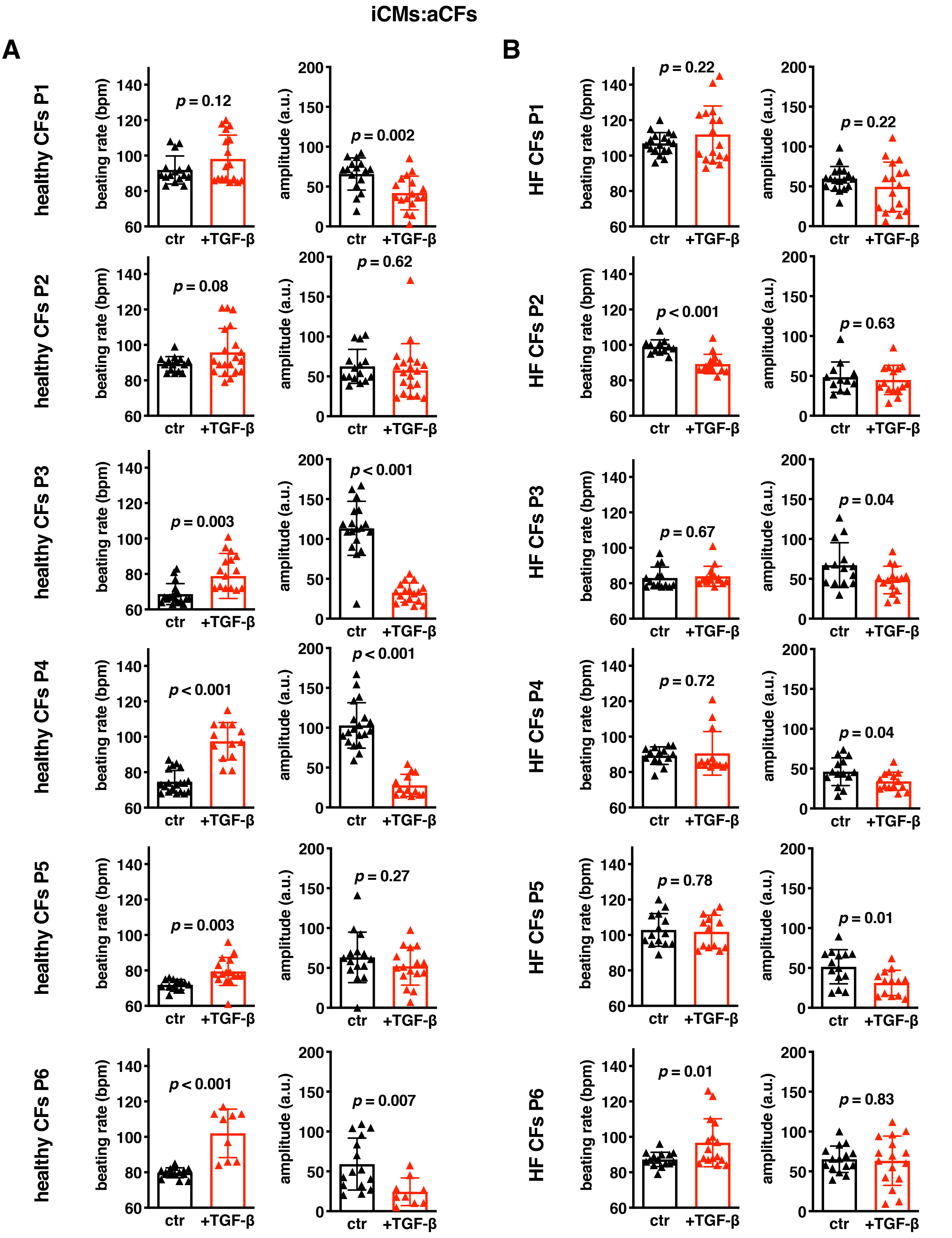

Supplementary Figure 11. Effect of exogenous TGF-β1 on contractility of healthy iCMs:aCFs and HF iCMs:aCFs microtissues. Figure shows quantification of contraction parameters of healthy iCMs:aCFs and HF iCMs:aCFs microtissues cultured in the presence (red) or absence (black) of TGF-β1 (10 ng/mL) at day 10. P1-P6 indicate aCFs from individual patients. Each dot represents data for one microtissue. *p* values were calculated with the Student's *t*-test.

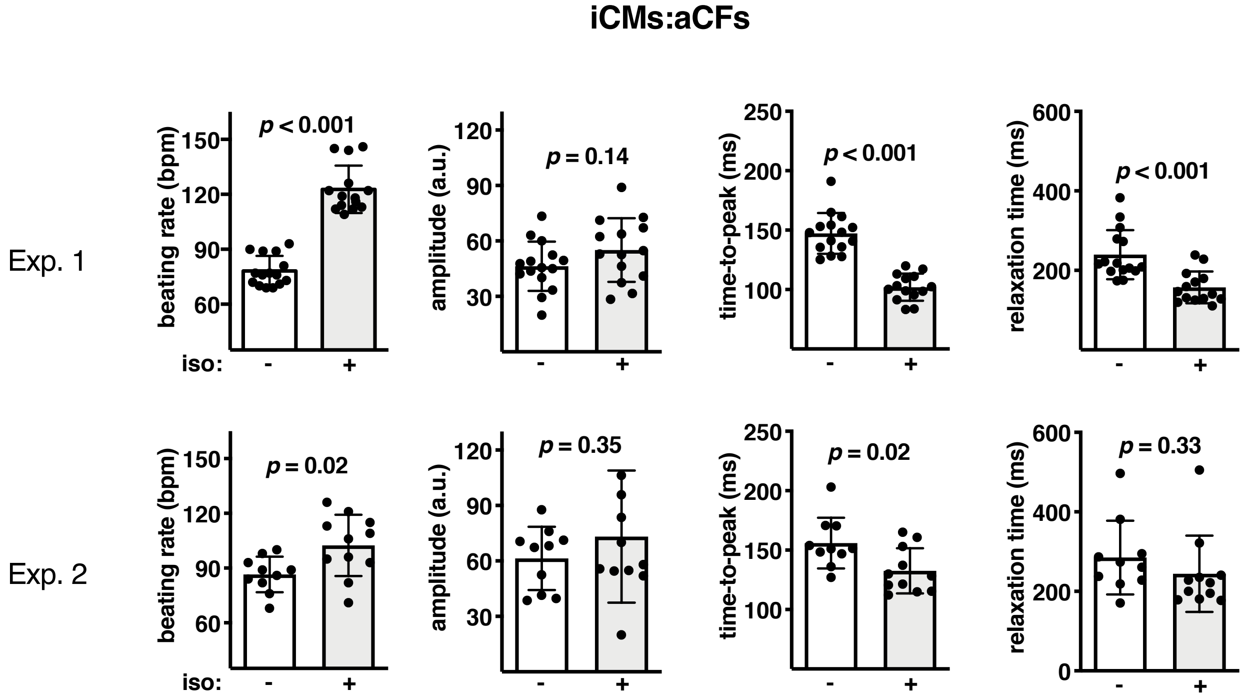

Supplementary Figure 12. β-adrenergic receptor stimulation of iCMs:aCFs microtissues. Quantification of contraction parameters of iCMs:aCFs microtissues stimulated with β-adrenoceptor agonist isoproterenol (iso, 10 nM). Unstimulated microtissues were used as controls. Each dot represents data for one microtissue. Graphs show data of 2 independent experiments. *p* values were calculated with the Student's *t*-test.

Supplementary Table 1. Clinical characteristics of patients from which aCFs were obtained. Patients no. 1-6 – healthy, patients 7-12 – heart failure.

Patient	Gender	Age	Ejection fraction	Clinical Diagnosis
**1**	male	16		No cardiovascular disease
**2**	male	22		No cardiovascular disease
**3**	male	12		No cardiovascular disease
**4**	male	14		No cardiovascular disease
**5**	male	11		No cardiovascular disease
**6**	male	23		No cardiovascular disease
**7**	male	51	20%	Myocardial infarction 6 years before, atrial fibrillation, hypertension
**8**	male	46	20-25%	Myocardial infarction 5 years before, atrial fibrillation
**9**	male	55	25%	Diabetes, myocardial infarction 8 years before
**10**	male	52	15%	Myocardial infarction 9 years before, hypertension
**11**	male	19	30%	Dilation of left atrium and left ventricle, mitral regurgitation
**12**	male	44	20%	Myocardial infarction 6 years before, diabetes, hypertension

Supplementary Table 2. Primers used for quantitative RT-PCR.

Gene	Forward (5' – 3')	Reverse (5' – 3')
**Vim**	GCTTCAGAGAGAGGAAGCCG	AAGGTCAAGACGTGCCAGAG
**COL1A1**	TTTTGTATTCAATCACTGTCTTGCC	CAGCCGCTTCACCTACAGC
**POSTN**	GAGACACACCCGTGAGGAAG	CACTGAGAACGACCTTCCCTT
**MMP2**	GTGCCTTCCAAGTCTGGAGCGATG	GGAGTCCGTCCTTACCGTCAAAG
**FN1**	GGAGAATTCAAGTGTGACCCTCATG	TGCCACTGTTCTCCTACGTGG
**FSP1**	CCACAAGTACTCGGGCAAAGAGG	CCTGTTGCTGTCCAAGTTGCTCATC
**FAP**	TGGAGATACTCTTACACAGCAAC	TGGACGAGGAAGCTCATTTCC
**TENC**	GGACCAAAACCATCAGTGCC	CCAGGAAACTGTGAACCCGT
**ACTA2**	ATGCCATGTTCTATCGGGTACTT	GACAATGGCTCTGGGCTCTGTAA
**COL3A1**	GGAATCTGTGAATCATGCCCTACTGG	ACCAGGATGACCAGATGTACCAGG
**IL11**	TCGAGTTTCCCCAGACCCTC	GAATCCAGGTTGTGGTCCCC
**MMP9**	AATCGCCAGTACTTCCCATC	CTTTGAGTCCGGTGGACGAT
**NPPB**	CAAGATGGTGCAAGGGTCTG	GACTTCCTCTTAATGCCGCCT
**MYH7**	GTAGACACACTTGAGTAGCCCA	TCTTGAGGTCAAAAGGCCTGG
**TNNI3**	GAGTCTCAGCATGGCGGATG	ATAAGCGCGGTAGTTGGAGG
**MYH6**	TCTCCGTGAAGGGATAACCAG	GCTCCTTCTCTGACTTGCGG
**NPPA**	GCAGGATGGACAGGATTGGAG	GTCCTCCCTGGCTGTTATCT
**MYL2**	TTGGGCGAGTGAACGTGAAA	AATGGTTTCCTCAGGGTCCG
**MYL7**	GGAGTTCAAAGAAGCCTTCAGC	TCCTCTGGGACACTCACCTT
**RPLP0**	ACACTGGTCTCGGACCTGAGAA	AGCTGCACATCACTCAGAATTTCA
